# Green Synthesized Silver Nanoparticles Using *Feijoa Sellowiana* Leaf Extract, Evaluation of Their Antibacterial, Anticancer and Antioxidant Activities

**DOI:** 10.22037/ijpr.2020.112523.13805

**Published:** 2020

**Authors:** Zahra Hashemi, Sobhan Mortazavi-Derazkola, Pouria Biparva, Hamid Reza Goli, Fereshteh Sadeghian, Mostafa Kardan, Alireza Rafiei, Mohammad Ali Ebrahimzadeh

**Affiliations:** a *Pharmaceutical Sciences Research Center, Hemoglobinopathy Institute, School of Pharmacy* *,* * Mazandaran University of Medical Sciences, Sari, Iran* *.*; b *Medical Toxicology and Drug Abuse Research Center (MTDRC), Birjand University of Medical Sciences, Birjand, Iran.*; c *Department of Basic Sciences, Sari University of Agricultural Sciences and Natural Resources, Sari, Iran. *; d *Molecular and Cell Biology Research Center, Hemoglobinopathy Institute, Faculty of Medicine, Mazandaran University of Medical Sciences, Sari, Iran.*

**Keywords:** Green synthesis, Anticancer, Antibacterial, Antioxidant, Feijoa sellowiana, Silver nanoparticles

## Abstract

Biogenic synthesis of silver nanoparticles (SNPs) has great attention of scientists, as it provides clean, biocompatible, non-toxic and inexpensive fabrication. In this study, *F. sellowiana* leaf extract was used for synthesizing SNPs which reduces silver nitrate into silver zero-valent. SNPs were characterized by UV, FTIR, XRD, SEM-EDS, and TEM analysis. They were also examined for their biological activities. The presence of biosynthesized SNPs was characterized by UV–visible spectroscopy and also crystal nature of SNPs was identified with XRD analysis. FT-IR spectrum was used to confirm the presence of different functional groups in the biomolecules which act as a capping agent for the nanoparticles. The morphology of SNPs was explored using SEM and the presence of silver was confirmed by elemental analysis. The size of the nanoparticles was in the range of 20–50 nm determined by TEM. The green synthesized SNPs showed good antibacterial activities against both gram-negative and gram-positive bacteria and also in resistant clinically isolated pathogens. Furthermore, the green synthesized SNPs showed reliable anticancer activity on the gastric adenocarcinoma (AGS) and breast (MCF-7) cancer cell lines with little effect on normal (HFF) cells. The *in-vitro* antioxidant activity of SNPs showed a significant effect on the scavenging of free radicals and iron chelating activity.

## Introduction

Nanotechnology implies characterizing and using material at the size of a nanometer in diverse fields consisting of biology, chemistry, physics, medicine, etc. which have attracted significant attention in recent years (1-3). Nano-medicine works at the same scale -around 100 nanometers or less than structures inside living cells and biological molecules function. Nano-medicines can play a significant role in developing alternative and more effective strategies for the diagnosis and treatment of malignancies, infectious and neurodegenerative disorders like Alzheimer’s disease (4, 5). Therefore, at the nanoscale, the nanomaterials because of their high surface-to-volume ratio display unique physicochemical effects that are not perceived in their bulk parent materials. The synthesis of metallic nanoparticles (NPs) has attracted significant interest due to their unique properties. In this regard, a variety of metals such as copper, magnesium, gold, zinc, titanium, and silver have been assessed that among them, the silver nanoparticles (SNPs) have exhibited a broad size distribution and morphologies with highly reactive aspects. Although several studies reported the genotoxicity effect of biogenic SNPs, most studies demonstrated their advantages for several human problems (6, 7). Many types of research have accomplished the antimicrobial activity of silver nanoparticles whose efficacy against parasites, viruses, fungi, and bacteria was accepted (8). 

According to several institutes studying cancer, it is anticipated that the incidence of cancer in the future will be extended to more than 20 million and there would be around 13 million deaths due to aging and population growth (9). Radiotherapy, chemotherapy, and surgery are some of the cancer treatments which are applied to make better the patients’ life. In addition, the side-effects of conventional treatment strategies caused major problems in the cancer treatment process. In spite of development in cancer control, morbidity and mortality rates are growing for all cancer types. Lately, the uses of Nanobiotechnology have discovered new strategies for the treatment and diagnosis of cancer (10, 11). Many reviews have discussed the original published articles to evaluate the efficacy of biologically synthesized nanoparticles against different types of cancer cells through *in-vitro* investigations (12). Anticancer activity of green synthesized SNPs has attracted interest because of their efficacy against cancer cell lines (13, 14).

Biological methods for the synthesis of nanoparticles can be devoted to applying the biological resources as nontoxic and eco-friendly reducing and stabilizing agents. The strategies for obtaining nanoparticles could be occurred by use of natural reagents, such as vitamins, sugars, plant extracts, biodegradable polymers, and microorganisms as both reluctant and capping agents which are attractive for nanotechnology (15, 16). These processes have led to making a few series of inorganic nanoparticles (importantly metal nanoparticles, metal oxides and salts). Through the reactant stated above, plant-based materials seem to be the best candidates and they are appropriate for the fabrication of nanoparticles via ‘biosynthesis’. Bioactive secondary metabolites such as phenols, flavonoids, alkaloids, and terpenoids are present in medicinal plants. These are used for curing many diseases including disorders and infectious diseases; they are also helpful in the green synthesis approach. Furthermore, capping agents are important for the stabilization of nanoparticles. Capped SNPs exhibit better activity compared to uncapped SNPs (17, 18). Synthesized green nanoparticles of biological sources like plants are widely accomplished because they are less toxic to humans compared with chemical medicines (15, 16). 

*Feijoa sellowiana* (Feijoa, Myrtaceae) is native to the south of America and is widely cultivated in Iran. Because of its high flexibility in subtropical regions, it is extensively cultivated in northern of Iran where its fruit is well known. Chemical compositions of this plant have been clearly reported but its pharmacological studies have been rarely carried out (19). It contains polyphenols, flavonoids, and vitamin C. A potent antimicrobial against *H. pylori* and anti-fungal activity was shown by Feijoa extract (20). We have recently reported its good antioxidant, nephroprotective (21), and antidepressant activities (22). Anti-toxoplasma effect (23), antibacterial and antifungal activities and strong inhibition of diabetes key enzymes (α-amylase and α-glucosidase) were reported from Feijoa (24). In addition, Feijoa has antitumor, gastroprotective, and hepatoprotective activities (25, 26). Its UV protective effect has been reported by our group (27). 

## Experimental

*Preparation of leaves extract of F. Sellowiana*

Feijoa leaves were collected from Fajr citrus experimental institute in May-June 2018. The plant was identified by Dr. Bahman Eslami. The voucher specimen (No. 194) was deposited in the herbarium of Sari School of Pharmacy. The leaves were dried at room temperature and roughly ground before extraction. One hundred grams of powder were extracted at room temperature by the maceration method. Methanol was used as an extracting solvent. The extracts were then separated from the sample residue by filter paper. The resultant extracts were concentrated in a vacuum by a rotary evaporator at 35 °C until crude solid extracts were obtained. For complete dryness, the crude extracts were freeze-dried. The yield was 36 g (24). 

*Synthesis of SNPs using F. sellowiana leaf extract*

 Silver nitrate (AgNO_3_) was purchased from Fluka Company. The other reagents were analytical grade and applied without more purification. In a typical reaction procedure, 0.1g crude extract was diluted to 100 mL distilled de-ionized water to make it 1 mg / mL and 12.5 mL of this solution were mixed with 12.5 mL, 4 × 10^−3 ^M aqueous silver nitrate solution. The mixture was heated at 65 °C with constant stirring for 30 min and silver nanoparticles were gradually obtained. Same reactions were also performed with various concentrations of AgNO_3_ in different temperature, pH within the different reaction times (28).

*Physicochemical characterization of SNPs*

UV–Vis absorption spectra were recorded on a spectrophotometer (T80+, China.) scanning from 300 to 600 nm. The crystalline structure of the SNPs was examined by X-ray diffraction, which was carried out using (Philips PW 1800). X-rays were generated at 30 kV and 10 mA. The XRD scans were recorded at 2θ from 10 to 90°. FTIR spectroscopy (Perkin Elmer Spectrum1) was used to scan the samples at a range 450-4000cm^-1^ of resolution to ensure the involvement of biomolecules, organic molecules responsible for the reduction of silver ions to SNPs. To study the micrograph images of synthesized SNPs, a scanning electron microscope (SEM, TESCAN BRNO-Mira3 LMU) was used. EDS was applied to examine the formation of synthesized SNPs using the (Ultima IV XRD system) scanning in 2θ (degree). DLS was used to assess the average hydrodynamic diameter (assayed by% of number) for the determination of the size of synthesized nanoparticles (Horiba-SZ-100-Z).TEM analysis was performed using a Zeiss EM-10C at an accelerating voltage of 100 kV.

*Cell viability study*

Two cancer cell lines including AGS (human gastric cancer cell line), MCF-7 (human breast adenocarcinoma cell line) and one HFF (human foreskin fibroblast) were attained from pasture Institute, Iran. The under investigation cells were grown in RPMI-1640. All of cell-cultured media were developed by 10% FBS and penicillin/ Streptomycin and incubated in humidified incubator at 37 °C in an atmosphere of 5% CO_2 _(11, 24).

The effect of nanoparticles on cellular proliferation was evaluated using MTT 3-(4, 5-dimethylthiazol-2-yl)-2, 5-diphenyltetrazolium bromide (Sigma, Germany) assay. The cells were plated in 96-well plates, 20,000 cells/well. Treatments were done after 24 h with solutions containing various concentrations of nanoparticles (SNPs) (3.12 and 1.56 μg/mL) three wells for each concentration. For each experiment, at least three wells were left untreated (control).

In presence of mitochondrial activity, MTT reagent was reduced to formazan crystals. To quantify cell bioactivity rate, formazan crystals were solubilized by adding DMSO. Then the absorption was carefully measured at 570 nm using Synergy H1 hybrid multi-model microplate reader (Biotek Instruments, Winooski, Vermont, NE, USA). The cell viability value of the silver nanoparticles was calculated by the following equation:

% cell viability = A_570_ of treated cells/A_570 _of control cells × 100%. 

Where A was the absorbance of sample or control. Cytotoxicity of silver nanoparticles is analyzed by using Graph pad prism 6 software.

*Antibacterial activities of synthesized SNPs*

Broth dilution method was performed for antibacterial tests by preparing serial dilution of SNPs in ranging from 75 to 0.75 µg/mL and 4000 to 500 µg/mL for Feijoa leaves extract in a 96-well microtitre plate. Muller Hinton broth was used as basal media and the bacterium were incubated at 37 °C for 24 h. MIC (minimum inhibitory concentration) and MBC (minimum bactericidal concentration) values of SNPs against the bacterial strains were determined according to method reported elsewhere (29). 100 mL of different concentration of SNPs which Muller Hinton Broth were used for dilution and 100 µL of bacteria (according to McFarland turbidity standards) of standardized microorganism suspensions were inoculated on to microplates and the test was performed in a volume of 200 µL. The Pplates were incubated at 37 °C for 24 h. MIC is determined as the lowest concentration of antibacterial agent that inhibits the visible growth of a microorganism. MBC is read as the lowest concentration of antimicrobial agent that kills all micro-organism, with complete absence of microbial growth (29).

Ciprofloxacin was used as reference compound for antibacterial activities at ATCC strains, the clinical isolated strains are resistance for several conventional antibiotics. *Staphylococcus aureus* (ATCC27853), *Acinetobacter baumannii* (ATCC29606) *Proteus mirabilis* (ATCC25933), *Enterococcus faecalis* (ATCC29213), *Escherichia coli* (ATCC25922), *Klebsiella pneumonia*(ATCC700603), and *Pseudomonas aeruginosa *(ATCC27853). All of the 7 isolated of bacteria from the patient’s sample (chip, urine, Blood peripheral, wound, phlegm) were received from the Department of Microbiology.

*Evaluation of Antioxidant activity of SNPs*

*free radical scavenging activity (DPPH assay)*

Antioxidant activity of synthesized SNPs was checked by DPPH (1, 1-diphenyl-2-picryl-hydrazil) method. In process, 1 mL of each concentration (60 to 3.75 mg/mL) of the synthesized SNPs was added to 1 mL DDPH solution and incubated in the dark at room temperature for 15 minutes subsequently, the absorbance of the mixtures was measured at 517 nm. Butylated hydroxyanisole (BHA) was applied as a positive control.

The ability of scavenging the DPPH radical was calculated using the following equation: 

DPPH Scavenged (%) = (Ac - As/Ac) × 100 

Where Ac and As are the absorbance of the control and test sample, A linear regression analysis was performed to determine the IC_50_ value (inhibitory concentration) for the sample (30).

*Fe* ^2+^ *chelating activity*

This method described that chelating agents are effective as secondary antioxidants because they reduce the redox potential, thus stabilizing the oxidized form of the metal ion. The iron-chelating activity of nanoparticles was evaluated using different concentrations of nanoparticles (9 to 200 µg/mL of SNPs). Thus, 3 mL of distilled water was added to 1 mL of SNPs and mixed with 50 µL of FeCl_2_ (2 mM) and 100 µL of ferrozine (5 mM). The mixture was shaken vigorously and left standing at room temperature for 10 min. Absorbance of the solution was then measured by UV-Vis spectrophotometer at 562 nm. The percentage inhibition of ferrozine Fe ^2+^ complex formation was calculated as

Ferrozine Fe^2 +^ complex % = [(A_0 _- As)/As] × 100 

Where A_0_ was the absorbance of the control, and As was the absorbance of the nanoparticles/standard. Na_2_EDTA was used as positive control (19).

## Results

**Figure 1:**
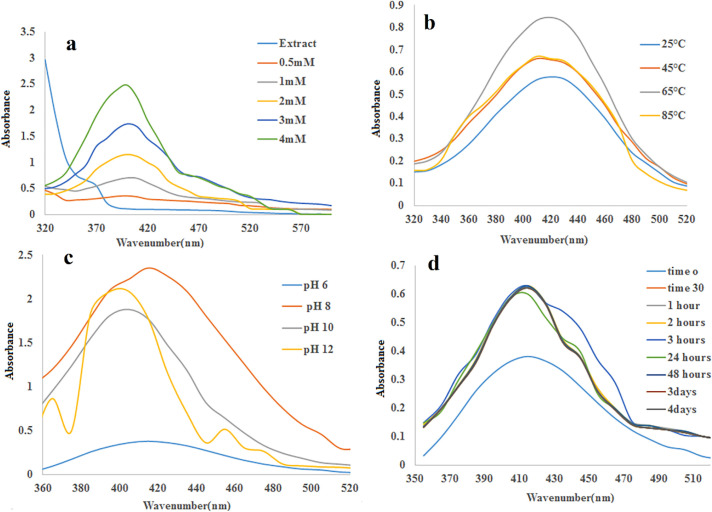
UV-visible spectrum of SNPs synthesis for optimization of (a) temperature (b) concentration of AgNO_3_ (c) pH (d) Time of reaction

**Figure 2:**
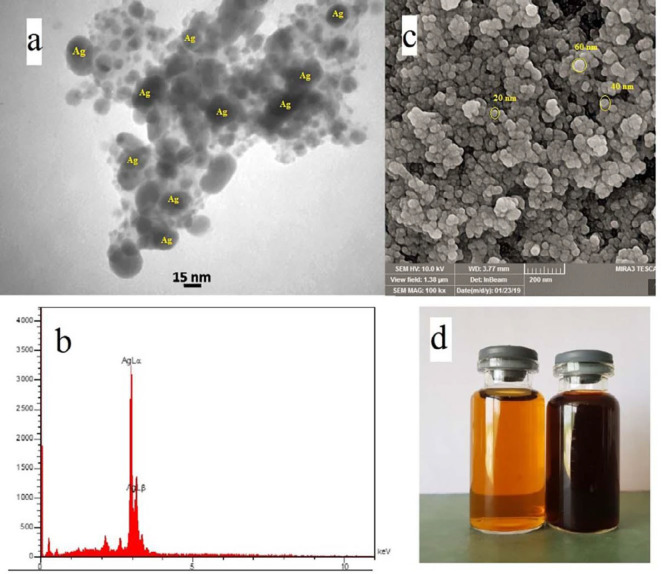
(a) TEM micrograph of the silver nanoparticles (b) EDS diagram (c) SEM micrograph at 200nm (d) Color changing after reaction which dark brown is synthesized SNPs

*Visual observation and UV-Vis spectroscopy*

(Figure 1a) shows the UV–visible spectra of silver nanoparticles formation using constant extraction concentration (1 mg/mL) with different concentrations of AgNO_3_ at room temperature. (Figure 1b) displays UV– visible spectra of the SNPs prepared at several temperatures. It can be seen that the absorbance increases with increasing temperature. (Figure 1c) shows the effect of pH on formation of silver nanoparticles. It can be observed that absorbance raises with increasing pH at 10 and then decreases. Furthermore, it is observed that the brown color of the nanoparticles emerged a little after mixing the AgNO_3_ with the extract (Figure 2d). The surface plasmon resonance (SPR) can be seen in UV-Vis spectrophotometer ranging 410–425 nm as absorbance peak. While the concentration of the silver nitrate enhances, the absorption peak gets more sharpness. The reaction between Ag^+^ and the reducing agents in the extract was monitored for four days, the UV–Vis spectra of Ag nanoparticles as a function of time was depicted at (Figure 1d) 

*Characterization of silver nanoparticles*

**Figure 3:**
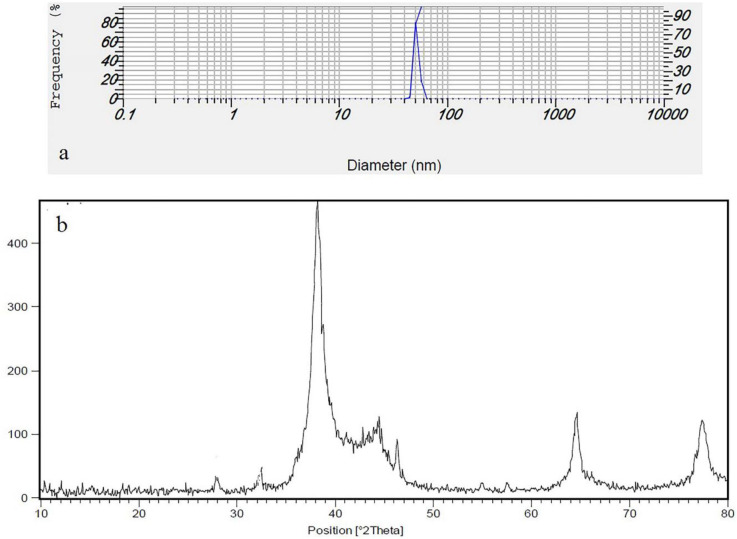
(a) DLS (b) XRD pattern of synthesis SNPs

**Figure 4:**
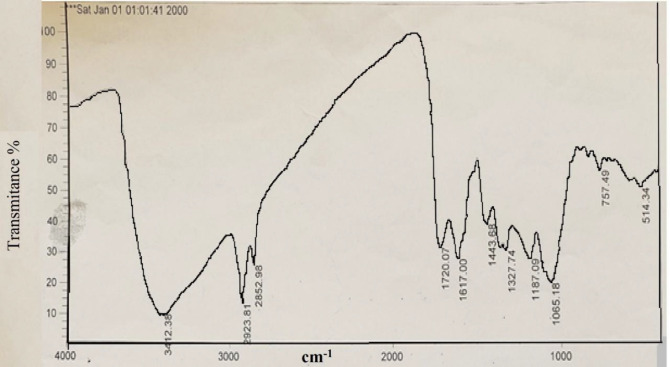
FTIR spectra of SNPs

In (Figure 2a) TEM image of silver nanoparticles derived from Feijoa leaf extract was shown. (Figure 2b) exhibited the EDX spectrum, that strong peak at 3eV confirms the formation of SNPs. In (Figure 2c). SEM technique was applied to visualize the size and shape of silver nanoparticles. The formation of silver nanoparticles as well as their morphological dimensions in the SEM evaluation proved that the average size was around 20-60 nm.

(Figure 3b) shows the XRD pattern of the prepared silver nanoparticles using Feijoa’s leaf extract. As can be seen in (Figure 3b), the diffraction peaks at 2θ = 38.26°, 44.1°, 63.8°, and 76.9° were related to Bragg’s from (111), (200), (220), and (311) planes, respectively. Two weak peaks at 2θ = 27.94° and 32.36° were attributed to impurity of AgCl. DLS is essentially used to determine particle size and size distributions in aqueous or physiological solutions. The results obtained for the samples are around 40 nm shown in (Figure 3a). In (Figure 4) FTIR analysis results of the SNPs, the prominent peaks were observed at 3412, 2923, 2852, 1720, 1618, 1443, and 1327 cm^−1^. Whereas 3412 cm^-1^ reveal bonded (–OH) hydroxyl group, the possible polyphenols in the leaf extract could be responsible for the reduction of the Ag + ions and capped them. On the other hand, the absorption peaks at about 2852 cm^−1^ could be specified to stretching vibrations of (-CH_2_) and (-CH_3_) functional groups. The peaks at 1720 and 1618 cm^−1^ show the fingerprint area of C = O, C-O, and O-H groups. The absorption band at 1443 cm^−1^ could be responsible on methylene scissoring vibrations.

*Antibacterial studies*

SNPs seem to be alternative antibacterial agents to antibiotics and have the ability to overcome the bacterial resistance against antibiotics. In this regard, the lowest MIC were observed for *S. aureus*,* P. aeruginosa*,* and A. baumannii* which were around 0.3, 0.3, 0.6 mg/mL and 9.5, 0.6, 4.5 mg/mL as MBC while being treated with SNPs. The results in (Table 1) shows that the synthesized silver nanoparticles have wonderful effect on inhibition of ATCC strains. Subsequently, for more comprehensive antibacterial effects SNPs were tested on isolated strains from the clinic which were resistant against several antibiotics. In our study seven clinical isolated bacteria were selected to examin susceptibility against synthesized SNPs. 

In this regard clinically isolate* S. aureus* from a catheter was susceptible for vancomycin; our synthesized SNPs showed the MIC of 1.17 mg/mL and the MBC of 9.37 mg/mL, respectively. *A. baumannii* and *P. aeruginosa* , isolated from wound and phlegm and the presence of the genes encoding the enzymes of the carbapenemase, were resistant against piperacillin/tazobactam, aztreonam, ceftazidime, cefepime, ciprofloxacin, levofloxacin, gentamicin, amikacin, tobramycin and imipenem, as well as doripenem and meropenem. Whereas carbapenems are the last line of treatment and the MIC for colestine sulfate was reported at 256 and 8 µg/mL respectively our synthesized SNPs significantly inhibited the growth of them at 0.58 µg/mL as MIC, 4.8 and 9.37 µg/mL as MBC.

Isolated* K. pneumonia* and* E. coli *from urine were resistant for carbapenems; the MIC for colistin sulfate were 128 and 32 mg/mL. But in our research the MIC were 9.37 and 0.58 mg/mL, correspondingly. Even though *P. mirabilis* isolated urine was resistant for imipenem and meropenem with the MIC of 2 and 32 mg/mL the synthesized SNPs showed the MIC value of 1.17 mg/mL with the MBC value of 9.37 mg/mL.* E. faecalis *which was isolated from peripheral was not susceptible for ampicillin, vancomycin, teicoplanin, erythromycin, tetracycline, levofloxacin, and kanamycin but was poorly susceptible for gentamicin. It could be inhibited by SNPs in the MIC value of 0.58 mg/mL and the MBC value of 9.37 mg/mL (Table 2).

*Anticancer activity and MTT assay*

**Figure 5:**
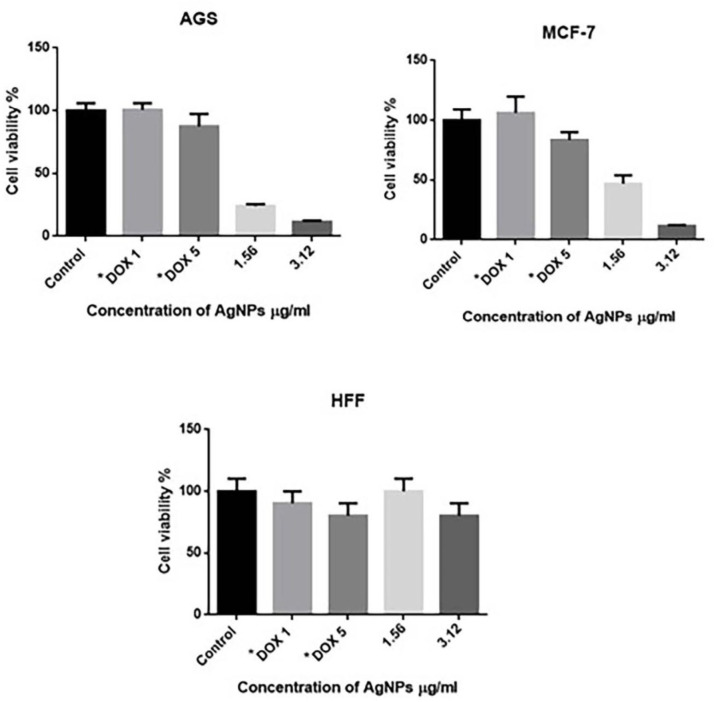
Cytotoxicity assay of the prepared SNPs against MCF-7, AGS, HFF cell lines *Doxorubicin 1mM and 5mM

 The *in-vitro* cytotoxic effects of SNPs were screened against AGS, MCF-7, and HFF cell lines and viability of tumor cells was confirmed using MTT assay. The viability of the cells (%) treated with various concentrations of SNPs has been shown in )Figure 5(. Cytotoxic effects of green synthesized SNPs were demonstrated in concentration of 3.12 mg/mL for both AGS and MCF-7 cell lines. Our results showed that the SNPs were more toxic for cancer cell lines (AGS and MCF-7) than for the HFF control (normal) cells.

*Antioxidant activity of SNPs*

*DPPH scavenging*

In current investigation, antioxidant activity of the biosynthesized silver nanoparticles using *F. sellowiana *leaf extract was studied which was found to be effective antioxidant in comparison with BHA as a standard antioxidant. With this method antioxidant activity of leaf extract of Feijoa have been assessed and estimated 80 mg/mL. In the present study, SNPs had the higher DPPH-scavenging activity (IC_50_ = 51.5 mg/mL) than their crud extract. Whereas BHA as standard had the IC_50_ value of 59 mg/mL, our nanoparticles could be introduced as a powerful free radical scavenger.

*Ferric chelating ability*

Iron chelating activity of *F. sellowiana *leaf extract has been reported as IC_50_ = 240 mg/mL. Results reveal that silver nanoparticle of Feijoa leaf extract exhibited effective ability for iron binding, proposing that its action as an antioxidant may be associated with its iron binding. Approximately our tested nanoparticles revealed Fe^2+^ chelating ability (IC_50_ = 214 mg/mL). EDTA displayed very strong activity (IC_50_ = 18 mg/mL) as standard.

## Discussion

*UV-Vis spectroscopy evaluation and characterization of SNPs*

Green nanotechnology is an interesting method for the synthesis of functional nanoparticles of gold, silver, zinc, and zero-valent iron (ZVINs), etc. (31, 32). Because of their wide application in biology, industrial processes, medicine, nanoscale materials have set up great attention in recent years. As a result, it is believed that the nanoparticles synthesis adopting biological principles is simple, safe, cost effective, and eco-friendly (33, 34).

Among various metal nanoparticles, silver nanoparticles (SNPs) have several effective applications in the field of biolabeling, sensors, antimicrobial, bactericidal activity against gram-positive, and gram-negative bacteria, including highly multiresistant strains such as methicillin-resistant *Staphylococcus aureus* (35). The use of plant extracts in green synthesis has been involved in various researches and studies. It was displayed that the production of metal nanoparticles using plant extracts could be accomplished in the metal salt solution in a few minutes at room temperature and the nature of the plant extract is an important factor. The concentration of extract, the metal salt, temperature, pH, and the contact time are significant affecting parameters. The synthesis of nanoparticles by means of plants contain several advantages like safety, availability and having a wide variety of active agents that can reduce silver ions. Biomolecules which operate as both reducing and capping agents and forming stable nanoparticles exist in ecofriendly plant extracts. Numerous biomolecules have been reported, such as phenolics, terpenoids, polysaccharides, flavones, alkaloids, proteins, enzymes, amino acids, and alcoholic compounds. The extract leaves, roots, latex, bark, stem, and seeds are used for nanoparticle synthesis. Flavonoids and phenols have distinctive chemical capacity to reduce and also effectively capp nanoparticles. They can be bind to metals via hydroxyl and carboxyl groups present in phenolic compounds (36).

UV-vis spectroscopy is a very valuable technique for the primary characterization of synthesized nanoparticles which are correspondingly used to screen the synthesis and stability of SNPs. In the present study, green synthesis of silver nanoparticles via plant intermediation was evaluated (37, 38). Our investigation proposes that the slow rate of SNPs production at room temperature can be enhanced by increasing temperature of the reaction mixture up to 65 °C. Absorbance peak rises with pH of extraction at 10. In previous researches, it was shown that the size and shape of biosynthesized nanoparticles could be influenced by changing the pH of the reaction mixtures. The major effect of the reaction pH, is its ability to vary the electrical charges of biomolecules which might affect their capping and stabilizing abilities and consequently the growth of the nanoparticles (39). The color of the solutions altered from light orange to dark brown depending on the extract concentration indicating silver nanoparticle formation as the color change observed is due to excitation of SPR in the silver nanoparticles (14, 40). This phenomenon happens when plasmonic metal like silver nanoparticle, have equal numbers of fixed positive ions in position and conduction mobile electrons. The irradiation of an electromagnetic wave led to driving the electric field to oscillate coherently called plasmons. Interaction of these plasmons with visible light is generated surface plasmon resonance (SPR). The SPR plays an important role in the assignment of optical absorption spectra of SNPs.  On the other hand, while the particle size increases, the absorption peak shifts to a longer wavelength. On the other hand, while the particle size increases, the absorption peak shifts to a longer wavelength. Increasing the reaction time resulted in increasing absorbance at 420 nm for up to one hour and being stable for four days. The intensity of the SPR peak increased as the reaction time increased, which indicated the increased concentrations of the silver nanoparticles. This result denotes that the silver nanoparticle provided by this green synthesis method is very stable without aggregation (41). The different shapes and sizes of SNPs caused some important physical and chemical properties. Synthesis procedures that make uniformly sized and shaped nanoparticles are being followed up. TEM is one of the improved techniques which evaluated the size and shape of the nanoparticles. It is noticeable that the majority of the TEM studies were done on plant extracted green synthesis of silver nanoparticles.

TEM analysis has revealed the presence of capping agents in the outer layer of plant-mediated nanoparticles. In this study, it was shown that SNPs synthesized using *F. sellowiana* leaf extract present a spherical morphology with a diameter ranging from 15 to 30 nm which involve a layer of extract as capping agent. EDX study has demonstrated the presence of silver in our synthesized nanomaterial (42, 43). The shapes of the silver nanoparticles were confirmed to be spherical using SEM micrograph. The SEM image shows the high density and homogeneity with the spherical morphology of silver nanoparticles synthesized by the *Feijoa* leaf extract. From incautious observation, it is apparent that the silver nanoparticles are surrounded by a thin layer of capping agent that is organic material from Feijoa leaf extract. (44,45). The X-ray diffraction pattern of the biosynthesized silver nanostructure produced by the leaf extract was further revealed and confirmed by the characteristic peaks observed in the XRD image (46). The size measured in DLS technique is the hydrodynamic diameter of the theoretical sphere that diffuses with the same speed as the measured nanoparticle. This size is not only associated with the metallic core of the nanoparticles but also influenced with all stuffs adsorbed on the outer layer of the nanoparticles such as reducer or stabilizers. The thickness of the electrical double layer and its effect on the measured size of nanoparticles hangs on the substances present on the surface of nanoparticles. As a result, the size measured in DLS technique is bigger in comparison with macroscopic techniques (47). In FT-IR evaluation we realized that these peaks are mainly attributed to phenolic compound present vastly in Feijoa leaf extract which is responsible in reduction of Ag ^+^ ions to Ag^0^ nanoparticles (48). 

*Antibacterial studies*

Since past centuries silver is broadly applied as an antimicrobial agent. Currently it is used as a potent antibacterial agent for wound dressing. Although antibiotics are widely employed in medical protocols, their long time exposure lets the bacteria become resistant. Resistance of bacteria occurred via different mechanisms which may be due to mutation in genes and formation of enzymes that inactivate antibiotics (49). Thus, antibiotic resistance in bacteria has caused a serious problem for scientists to concern this issue finding out antimicrobial activity of NPs is a new strategy to overcome this challenge. Amongst numerous NPs, silver is the most potent antibacterial agent. The antibacterial activity of SNPs depends on their size and shape. In several study it was observed that SNPs inhibited the bacterial pathogens in dose-dependent manner. Overall, with increasing SNPs concentration, the inhibition of bacterial pathogens gets more (50). In the current investigation, the antibacterial effect of SNPs at different concentrations (75 to 0.14 mg/mL) was quantitatively assessed on the basis of MIC and MBC value (48). In comparison with conventional antibiotics, SNPs exhibited strong antibacterial activity against all human pathogens. These results were observed for both ATCC and clinical isolated bacteria (51). SNPs have appeared as antibacterial agents against bacterial pathogens because of their high surface-area-to-volume ration and unique chemical and physical properties. Along with a decrease in the size of nanoparticles, the surface area-to-volume ratio of NPs increases. The small particle size allows nanoparticles to attach to the cell wall and penetrates into the bacteria cell, which makes possible their antimicrobial activity against bacteria (52). This activity against bacteria is related to the release of the silver ions which changes the membrane structure of the cell. As a result, permeability of the bacteria increases, and finally, cell death happens (47).

*Anticancer studies*

MTT assay was applied to evaluate the cytotoxicity of SNPs against breast cancer and gastric carcinoma along with normal cell lines. Interestingly, most of the studies described spherical SNPs with an average diameter of less than 100 nm which represented significant toxic effects with the inhibitory concentration of 50% in breast cancer cell lines, while less toxicity was reported in normal cell lines (11, 12). 

Remarkably, during the specification of target receptors on tumor cells metallic nanoparticles could be attached to biological fragments, such as tumor markers, monoclonal antibodies, peptides, etc. Moreover, it is highly important to notice that the properties of SNPs, such as aggregation of particles, size and shape, surface area, purity, solubility, capping agent, surface charge, and structural alteration are prominent properties affecting their cytotoxicity capacity (11, 53).

This study strongly discovered the significant anti-proliferative activity of biosynthesized silver nanoparticles with poor effect on normal cells when compared to the control (54). Cell inhibition of silver nanoparticles was reported at 3.043 mg/mL for MCF-7 cell line; in some studies concentration of 50 and 20 mg/mL also has been observed (55, 56). According to the reported results, it is concluded that Ag nanoparticles could have induced the generation of reactive oxygen species (ROS). When the NPs enter the cells, the ROS interact with the cellular materials and cause DNA damage and/or the mitochondria-dependent apoptosis pathway leading to cell death (56, 57). One hundred mg/mL was observed for AGS cell line while in the other study 10 mg/mL of silver nanoparticles was reported for cytotoxicity effect of green synthesized silver nanoparticles (58).

*Antioxidant activity of SNPs*

In a free radical scavenging study, a freshly prepared DPPH solution (40 ppm) displayed a dark purple color with maximum absorption at 517 nm. This purple color disappears when an antioxidant is existing in the medium. Thus, antioxidants molecules can reduce DPPH free radicals and alter them to a colorless product. These biosynthesized nanoparticles ranging (60, 30, 15, 7.5, and 3.25 mg/mL) concentration, showed antioxidant activity and DPPH values were increased in a dose dependent manner (59). The main aim of avoiding ROS generation is related to redox-active metal catalysis; this subject happens by chelating the active metal ions. The chelating of Fe^2+ ^by green synthesized silver nanoparticles was estimated. Fe^2+^ could form complexes with Ferrozine quantitatively. The red color of the complex is diminished while the chelating agent exists in the medium and the complex formation is disturbed with that result. The amount of color reduction lets the estimation of the chelating activity of iron (19,30). In our study, we were able to exhibit this potential with our green synthesized nanoparticles.

**Table 1:** Antibacterial activities of NPs at ATCC bacteria strain

**MBC (g/mL)**	**MIC (g/mL)**	**MIC of extract** **(g/mL)**	**Ciprofloxacin** **MIC (g/mL)**	**ATCC**	**Bacteria**
9.5	0.3	>4000	0.21	ATCC 29213	*S.aureus*
37	18	>4000	0.21	ATCC 29212	*E.faecalis*
4.5	0.6	>4000	512	ATCC 27853	*P.aeruginosa*
0.6	0.3	>4000	0.25	ATCC 19606	*A.baumannii*
9.5	0.6	>4000	0.1	ATCC 25922	*E. coli*
9.5	0.6	>4000	0.1	ATCC 700603	*K.pneumoniae*
4.5	0.6	>4000	0.1	ATCC 25933	*P.mirabilis*

**Table 2:** Antibacterial activities of NPs at clinically isolated and resistant strains

**Susceptibility to antimicrobial agents**											**Treatment with NPs**		**Bacteria**
**MC**	**VC**	**OC**	**TC**	**GM**	**PC**	**CIF**	**CEZ**	**ERM**	**CM**	**AK**	**MIC SNPs** **(µg/mL)**	**MBC SNPs (µg/mL)**	
R	S	R	R	R	R	R	R	R	R	R	1.17	18.75	*S. aureus*
R	R	R	R	S	R	R	R	R	R	S	0.58	9.37	*E. faecalis*
R	R	R	R	R	R	R	R	R	R	R	0.58	4.8	*P. aeruginosa*
R	R	R	R	R	R	R	R	R	R	R	0.58	9.37	*A. baumannii*
R	R	R	R	R	R	R	R	R	R	R	0.58	18.75	*E. coli*
R	R	R	R	R	R	R	R	R	R	R	9.37	150	*K. pneumoniae*
R	R	R	R	R	R	R	R	R	R	R	1.17	9.37	*.* *P.mirabilis*

**MC**, Meticillin; **VC**, Vancomycin; **OC**, Oxacillin; **TC**, Tetracycline; **GM**, Gentamicin; **PC**, Penicillin; **CIF, **Ciprofloxacin; **CEZ**, Ceftazidime; **ERM**, Erythromycin; **CM**, Clindamycin; **AK**, Amikacin

## Conclusion

The facile green procedure for synthesis of silver nanoparticles was done in the current study using *F. sellowiana* leaf extract. The green synthesized nanoparticles were characterized by different tools. The SPR peak at 420 nm in UV–Vis spectra was primarily characterized and the creation of SNPs was confirmed. Using the UV-Vis spectroscopy, the condition of reaction was optimized and the stability of the biosynthesized nanoparticles was evaluated. From FTIR analysis it was confirmed that the extract was multifunctional, which was as reducing and also stabilizing agent. In addition, TEM, SEM, EDS, and XRD characterization discovered the creation of crystalline spherical nanoparticles with average particles size of 30 nm. This article represented that nano-biotechnology has the potential to inhibit and kill several bacterial pathogens and also combat with breast and gastric tumor cells; however, the studies in this area are still in laboratory-type setting and many challenges should be considered. SNPs can be introduced as a potent anticancer agent against MCF-7 and AGS cell lines which make them a good candidate for chemotherapy processes. Bio-synthesized SNPs is offering a potent antioxidant along with their iron chelating activity which can introduce a new vision for study and treatment of disease related with iron-overload problems.
